# The Quest for the Identification of Genetic Variants in Unexplained Cardiac Arrest and Idiopathic Ventricular Fibrillation

**DOI:** 10.1371/journal.pgen.1003480

**Published:** 2013-04-11

**Authors:** Pieter G. Postema

**Affiliations:** Department of Cardiology, Heart Center, Academic Medical Center, University of Amsterdam, Amsterdam, The Netherlands; Georgia Institute of Technology, United States of America

## Unexplained Cardiac Arrest

**Figure 1:**
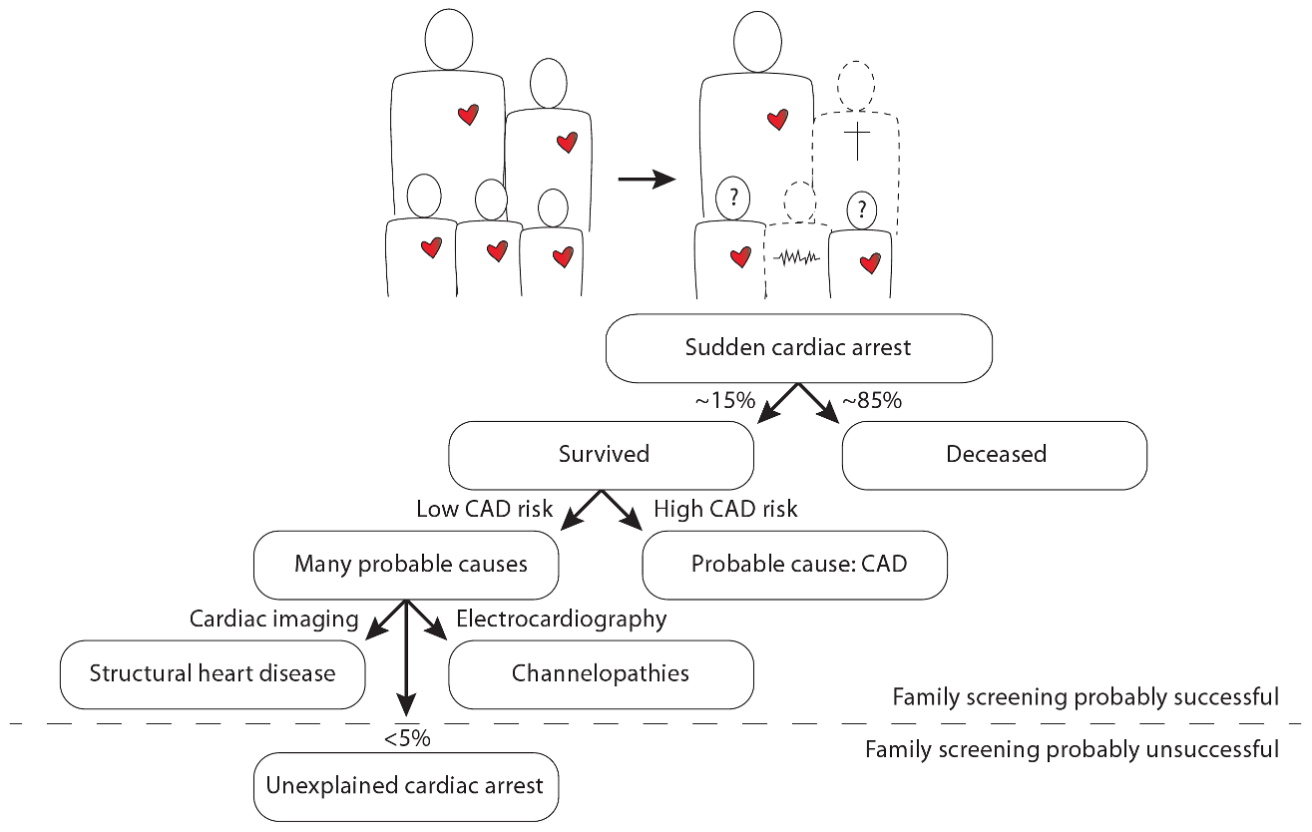
Illustration of the position of unexplained cardiac arrest among sudden cardiac arrest, focused on the occurrence of familial sudden cardiac arrest. The question marks denote the uncertainty in the remaining siblings as to whether they too have an increased risk for sudden cardiac arrest and whether it is possible that they transmit this risk to their own offspring. CAD denotes coronary artery disease.

In this issue of *PLOS Genetics*, Nakano and colleagues from 13 centers in Japan report a candidate gene analysis in unrelated individuals with an unexplained cardiac arrest (UCA) [1]. Cardiac arrest is most often caused by a cardiac arrhythmia named ventricular fibrillation, which, if left untreated, will be lethal within minutes. Moreover, ventricular fibrillation is often the first expression of the disease, implying that patients die suddenly without any warning. It is thus understandable that cardiac arrest is an extremely difficult phenotype, as its victims will not be pre-symptomatically treated and the vast majority (∼85%) will not survive the event. In those few who do survive, the sequelae to cardiac ischemia in coronary artery disease appear to be the predominant cause of the cardiac arrest (i.e., the direct pro-arrhythmic effects of ischemia as well as the indirect pro-arrhythmic effects of successive cardiac remodeling and fibrosis). Several genetic variants that predispose to cardiac arrest in the setting of cardiac ischemia have already been documented [2], [3]. In the absence of coronary artery disease or other clear causes (e.g., myocarditis), structural heart disease (e.g., hypertrophic cardiomyopathy) and channelopathies (e.g., long QT syndrome, Brugada syndrome) are now recognized as the most prevalent causes of cardiac arrest, especially in younger individuals [4]. Nevertheless, in some individuals (<5%) the cause of the cardiac arrest remains unexplained: these unexplained cases comprise different, yet unidentified, pathologies (Figure 1).

Importantly, UCA appears to have a heritable component in several patients [5], which may thus set their whole family at risk for premature sudden death. A number of UCA cases with documented ventricular fibrillation can be defined as having idiopathic ventricular fibrillation (IVF). This more strict definition is reserved for those patients who survive ventricular fibrillation and in whom all extensive evaluations return normal. The first, and only, breakthrough in defining the genetic underpinnings of IVF was made in 2009 in The Netherlands [5]. Genome-wide haplotype analysis in several related IVF families revealed a conserved haplotype in the *DPP6* gene, putatively involved in one of the main cardiac potassium currents: the transient outward current. This discovery has already led to the identification and pre-symptomatic treatment of dozens of patients at risk for IVF. However, mutations in the *DPP6* gene have not yet been implicated in IVF/UCA outside The Netherlands.

This continuing lacuna hampers both treatment and cascade screening for the identification and treatment of affected family members and results in both under-treatment and over-treatment with substantial collateral damage (including mortality). Hence, UCA is both an uncommon and a difficult phenotype, and it represents a bothersome black box in current cardiology. Further efforts to delineate its underlying genetic profile will help us to understand this entity and, importantly, will hopefully guide us to the identification of as yet pre-symptomatic but similarly affected family members.

## *Semaphorin 3A* and Unexplained Cardiac Arrest

Nakano and colleagues studied two populations with UCA and documented ventricular fibrillation. In east and west Japan, they retrospectively collected cases who survived UCA (*n* = 31 and *n* = 52, respectively) and controls (*n = *912 and *n* = 2,046, respectively) from several cohorts. Guided by their previous results in mice studies [6], they focused on *SEMA3A* as a candidate gene. *SEMA3A* is vital for normal neuronal pattern development, and has been implicated in various disease conditions [7], [8]. Interestingly, murine cardiac-specific *SEMA3A* under-expression results in sinus bradycardia and *SEMA3A* over-expression in a susceptibility to ventricular tachycardia and sudden death, putatively due to differences in the pattern of cardiac innervation [6]. In their current study, through resequencing and SNP genotyping of *SEMA3A*, it was found that a nonsynonymous polymorphism (I334V, rs138694505A>G) in exon 10 of the *SEMA3A* gene was associated with UCA with an odds ratio of 3.1 (95%CI 1.7–5.7). UCA patients with *SEMA3A*I334V also displayed slightly different autonomic nervous system control, apparently as a result of a higher incidence of sinus bradycardia. Subsequent *in vivo* studies using cardiac biopsies revealed that UCA patients with *SEMA3A*I334V display aberrant sympathethic nerve fiber growth. Furthermore, *in vitro* studies using transfected HEK293T cells revealed that the I334V amino acid replacement in SEMA3A indeed disrupts the normal function of the protein in neural growth inhibition and control of cardiac innervation.

## Strengths and Limitations

The strengths of the current study are several. The authors were able to unite data from the extremely rare but extremely challenging UCA cases who survived documented ventricular fibrillation from many different centers in Japan, and they replicated the association between UCA and *SEMA3A*I334V in two study groups (east and west Japan). Further clinical, *in vitro*, and *in vivo* studies substantiated their findings. These results, in combination with the previous studies in *SEMA3A* mice [6] and other studies, make a strong argument for a pivotal role of *SEMA3A* in cardiac innervation patterning and, if that goes wrong, UCA.

However, there are several limitations that should be acknowledged. Although it can be expected that cases with such a rare phenotype are difficult to assemble, the low numbers by current genetic association standards clearly prohibit definitive conclusions. Another important limitation is the lack of segregation data in the families of the victims. To be able to use *SEMA3A*I334V as a risk stratifier for sudden death in the remaining pre-symptomatic family members (which is the ultimate goal), one should have indisputable evidence that inheritance of the risk allele is the predominant cause of the increased risk for sudden death in these families. Only with this knowledge can one easily justify aggressive pre-symptomatic therapy such as implantation of a cardioverter defibrillator. However, as the family members of the studied cases apparently refused screening during this study, the clinical usefulness of *SEMA3A*I334V currently remains unknown.

Furthermore, this phenotype of UCA with documented ventricular fibrillation evidently has several different underlying causes, including IVF but also cardiomyopathies/channelopathies of unknown origin, as can be discerned from the electrocardiograms (Figure 2 from Nakano et al. [1]). It could be suggested that *SEMA3A* plays a role in all of these different underlying causes, but it is more likely that certain phenotypes will be more affected by *SEMA3A* dysregulation than others. It should be noted that 85% of *SEMA3A*I334V patients underwent a pilsicainide provocation test, which excluded Brugada syndrome in these patients (supplementary Table S2 from Nakano et al. [1]). This suggests that *SEMA3A*I334V does not result in a Brugada phenotype. However, it is believed that arrhythmias in Brugada syndrome are exacerbated by vagal stimulation, which might suggest *SEMA3A* as a candidate gene for future studies of Brugada syndrome.

The quest for the identification of genetic variants in UCA and IVF will certainly continue and meticulous phenotyping of the patients will remain an important issue. Genome-wide studies in unrelated individuals are unlikely to be revealing for the near future, but Nakano et al. demonstrate the ongoing value of candidate gene studies. More study populations are needed that are suitable for discerning phenotype–genotype relationships, which then can be further analyzed in translational studies.

## Conclusions

Nakano and colleagues studied the rare and difficult but important phenotype of UCA, and revealed that *SEMA3A*I334V is associated with a higher incidence of cardiac arrest. These results were substantiated by *in vitro* and *in vivo* studies and point to a pivotal role for *SEMA3A* in cardiac innervation patterning and, if that goes wrong, a propensity for sudden cardiac arrest.
